# Effects of compound Danshen dropping pills on adverse cardiovascular events and quality of life after percutaneous coronary intervention in patients with coronary heart disease: A protocol for systematic review and meta-analysis

**DOI:** 10.1097/MD.0000000000028994

**Published:** 2022-02-25

**Authors:** Lina Lv, Xianying Yuan, Lihong Jiang

**Affiliations:** a Changchun University of Chinese Medicine, Changchun, Jilin, China; b Department of First Clinical Nursing, School of Nursing, Changchun University of Chinese Medicine, Changchun, Jilin, China; c Affiliated Hospital of Changchun University of Chinese Medicine, Changchun, Jilin, China.

**Keywords:** adverse cardiovascular events, compound Danshen dropping pills, meta-analysis, percutaneous coronary intervention, protocol

## Abstract

**Background:**

Percutaneous coronary intervention (PCI) is an important means for the treatment of coronary atherosclerotic heart disease and has effectively reduced the mortality of coronary heart disease. However, reperfusion can also cause certain damage to the vascular endothelium, leading to the major adverse cardiovascular events. Compound Danshen dropping pill is a Chinese patent medicine preparation. At present, many studies have evaluated the effect of compound Danshen dropping pill in reducing the incidence of adverse cardiovascular events after PCI. This study systematically evaluated the effect of compound Danshen dropping pills on major adverse cardiovascular events and quality of life after PCI and provides a reference for clinical application and research.

**Methods:**

All randomized controlled trials on the effects of compound Danshen dropping pills on adverse cardiovascular events and quality of life after PCI for coronary heart disease were searched for. The search was conducted from database inception to January 2022. Data extraction and quality assessment were performed by 2 reviewers according to the Protocol Guidelines for Systematic Reviews and Meta-analyses Protocols statement guidelines. Meta-analysis was performed using Review Manager Version 5.4 software and Stata 16 software.

**Results:**

The results of this study will allow for systematically evaluation of the effects of compound Danshen dropping pills on adverse cardiovascular events and quality of life after PCI for coronary heart disease.

**Conclusion:**

This study will provide objective evidence of a basis for compound Danshen dropping pills reducing adverse cardiovascular events and improving quality of life after PCI.

## 1 Introduction

Coronary atherosclerotic heart disease (CHD) is a heart disease caused by myocardial ischemia, hypoxia or necrosis due to stenosis, spasm or blockage of the coronary artery lumen.^[1]^ It has become the leading cause of death recognized by the World Health Organization (WHO).^[2,3]^ Percutaneous coronary intervention (PCI) is an important treatment for coronary heart disease that can quickly and effectively open occluded blood vessels and restore the blood oxygen supply of the myocardium, with less trauma, a short course of treatment, and remarkable effects. At present, PCI has been widely used in the clinic setting and effectively reduces the mortality of CHD.^[4]^ However, in clinical practice, there are also some problems in PCI treatment. PCI cannot eliminate the risk factors of CHD, nor can it reverse the development of coronary atherosclerosis. At the same time, when the infarcted blood vessel is opened, it will also cause some damage to the vascular endothelium, leading to the occurrence of major adverse vascular events such as restenosis in the stent, recurrence of angina pectoris, arrhythmia, secondary myocardial infarction, revascularization, heart failure, and even cardiogenic death, which has brought adverse effects to the prognosis of patients.^[5]^ At present, conventional Western medicine treatment after PCI has achieved certain curative effects, but the incidence of major adverse cardiovascular event (MACE) is still high, so it is remains important to discuss the related treatment management of patients after PCI.

Compound Danshen dropping pill is a Chinese patent medicine preparation mainly composed of Danshen, Sanqi, and Borneol. Modern pharmacological research shows that it has the functions of antiplatelet aggregation, reducing inflammatory reactions and vascular endothelial injury.^[6]^ Compound Danshen dropping pills can effectively relieve the clinical symptoms of patients after PCI and improve the clinical efficacy, but there is no relevant systematic review published yet. Therefore, this study systematically evaluates the influence of compound Danshen dropping pill on adverse cardiovascular events and quality of life after percutaneous coronary intervention for coronary heart disease and further collects evidence to provide a medical reference for clinical research.

## 2 Methods

### 2.1 Study registration

The study has been registered on INPLASY (https://inplasy.com/inplasy-2022-1-0044/), registration number: INPLASY202210044. This protocol will be strictly implemented in accordance with the system evaluation and meta-analysis protocol (PRISMA-P).^[7]^

### 2.2 Inclusion criteria

#### 2.2.1 Types of studies

We collect all randomized controlled trials on the effects of compound Danshen dropping pills on cardiovascular adverse events and quality of life after percutaneous coronary intervention. The included documents were not restricted by blinding or distribution hiding requirements, but the languages of the literature were limited to Chinese and English.

#### 2.2.2 Participants

We include patients who meet the diagnostic criteria of coronary heart disease and receive percutaneous coronary intervention. There is no restriction on age, sex, course of disease, or race.

#### 2.2.3 Interventions

The control group must have received routine treatment. On the basis of routine treatment, the experimental group was treated with compound Danshen dropping pills. The treatment time is after PCI, and the dosage and course of treatment is not limited. Routine treatment includes antiplatelet drugs, anticoagulants, nitrates, beta blockers, calcium channel blockers, lipid-lowering drugs, and so on.

#### 2.2.4 Outcomes

The primary outcome measure was adverse cardiovascular events, including recurrent angina, severe arrhythmia, heart failure, nonfatal myocardial infarction, repeat revascularization, in-stent restenosis, and cardiac death. The secondary outcome measures include high sensitivity C-reactive protein, left ventricular ejection fraction, 6-minute walking test, and the Seattle Angina scale (degree of physical activity limitation, angina stable state, angina attack, treatment satisfaction, and disease awareness).

### 2.3 Exclusion criteria

The data are incomplete or there are obvious errors, and so statistical analysis cannot be carried out. Research on intervention measures containing traditional Chinese medicine (TCM) preparations similar to compound Danshen dropping pills. Repetitively published literature. The full text is unable to be obtained through either electronic or manual retrieval.

### 2.4 Search strategy

We selected 7 databases, including PubMed, the Web of Science, Embase, Cochrane Library, the Chinese National Knowledge Infrastructure, the Chinese Science Journal Database, Wanfang Data, and the Chinese Biomedical Literature Database, for retrieval. We searched for the effects of compound Danshen dropping pills on cardiovascular adverse events and quality of life after percutaneous coronary intervention. The search time was from database establishment to January 2022. Search only Chinese and English literature. The database search was carried out in the form of subject headings combined with free words. The search terms included “Compound Danshen Dropping Pills”, “Percutaneous Coronary Intervention”, “Standard Balloon Angioplasty”, and “Intracoronary Insertion”. In addition, references to the included literature were traced back to supplement the acquisition of relevant literature.

### 2.5 Study selection and data exaction

The literature screening was independently performed by 2 researchers. First, by reading the title and abstract of the literature, literature that did not meet the standard was initially screened. After reading the full text, the second screening was conducted according to the inclusion and exclusion criteria. In case of disagreement, both parties discussed or consulted a third party for judgment. Data were extracted independently by the 2 researchers, including the name of first author, publication time, title of paper, name of disease, sample size of each group, intervention time, intervention method, outcome indicators, and bias risk evaluation. After completion of the cross-check between the 2 investigators, those with inconsistent results were discussed, or the third investigator was consulted to reach a consensus.

### 2.6 Risk of bias assessment

The risk of bias in the included studies was evaluated by 2 researchers using the Cochrane Systematic Evaluator's Manual 5.1.0 bias risk assessment tool.^[8]^ Evaluation criteria include random method selection; allocation hiding; blind method, completeness of the result data; whether the evaluator is blind; selectively reporting results; Other Biases 7 entries. According to the specific criteria of the evaluation manual, the researchers identified the included studies as low risk bias, high risk bias or unclear risk of bias. Disagreements were resolved through consultation with a third party.

### 2.7 Data synthesis and analysis

#### 2.7.1 Data synthesis

Quantitative comprehensive analysis was performed using the RevMan version 5.4 software (The Cochrane Collaboration, Copenhagen, Denmark). For continuous variables, the mean difference (MD) and standardized mean difference (SMD) were used as effect indices, whereas for dichotomous variables, the relative risk (RR), odds ratio (OR), and risk difference (RD) were used as effect indices. A 95% confidence interval was used as the effect size, and *P* ≤ .05 was considered statistically significant.

#### 2.7.2 Assessment of heterogeneity

A chi-square test was used to analyze the heterogeneity among the included studies. If *P* > .1 and *I*^2^ < 50%, it is determined that there was no statistical heterogeneity among the included studies, and a fixed effect model could be used. If *P* < .1 and *I*^2^ ≥ 50%, a random-effect model was selected to analyze the source of heterogeneity (methodological heterogeneity, clinical heterogeneity), and subgroup or sensitivity analysis could be performed. If the source of heterogeneity cannot be determined, only descriptive qualitative analysis can be used.

#### 2.7.3 Sensitivity analysis

By changing the combined effect size model, excluding some low-quality, high-weight studies, or excluding each included study individually and then merging the effect size. Compared with the consolidated statistics before and after elimination, if there was no significant change before and after elimination, the results of the meta-analysis were robust and credible. In contrast, the meta-analysis showed that the sensitivity was high and the robustness of the results was low, so we should be cautious in interpreting the results, drawing conclusions, and further clarifying the source of bias.^[9]^

#### 2.7.4 Reporting biases assessment

If the number of included studies for the same outcome was ≥10, a funnel plot was used to assess publication bias.^[10]^

#### 2.7.5 Missing data management

If relevant data were missing, we contacted the author of the article by phone or email to attempt to obtain them.

### 2.8 Ethics and dissemination

All data in this study are from published studies, no patient recruitment is required, no personal privacy is involved, and therefore no ethics committee approval is required.

## 3 Discussion

With the acceleration of population aging and changes in people's living habits, the morbidity and mortality of cardiovascular diseases continue to rise, seriously endangering people's health.^[11,12]^ In recent years, the widespread adoption of percutaneous coronary intervention has benefited many CHD patients. However, PCI may cause the occurrence of MACEs, such as in-stent restenosis, angina pectoris recurrence, repeated myocardial infarction, and even cardiogenic death, which leads to the clinical symptoms of patients being impossible to alleviate or even life-threatening. There are many factors that cause postoperative MACE, and the conventional Western medicine recommended by the guidelines is often aimed at only 1 or a few mechanisms, which is ineffective and will bring about adverse reactions.^[13]^

TCM has received increasing attention for the prevention and treatment of post-PCI complications. Based on conventional Western medicine, the combination of TCM adjuvant therapy can coordinate with Western medicine to regulate cardiac function in multiple ways and multiple targets and inhibit the occurrence and development of MACE, thus improving the prognosis of patients and improving the quality of life.^[14]^ Compound Danshen dropping pills are widely used in the treatment of coronary heart disease. The drug can expand the coronary artery, improve coronary flow, increase myocardial blood oxygen supply, improve myocardial ischemic injury, and relieve symptoms of cardiac dysfunction. This study collected and analyzed clinical research data on the effect of compound Danshen dropping pills on MACEs and quality of life after PCI, with the main purpose of providing guidance for clinical application and research.

## Author contributions

**Conceptualization:** Lina Lv, Lihong Jiang.

**Data curation:** Xianying Yuan.

**Formal analysis:** Lina Lv.

**Funding acquisition:** Lihong Jiang

**Investigation:** Xianying Yuan.

**Methodology:** Lina Lv, Xianying Yuan.

**Project administration:** Lihong Jiang

**Resources:** Lina Lv.

**Software:** Lina Lv.

**Supervision:** Lihong Jiang.

**Validation:** Lihong Jiang.

**Visualization:** Xianying Yuan.

**Writing – original draft:** Lina Lv.

**Writing – review & editing:** Lina Lv.

## References

[1] Kaluski E Waller A Patel A. Clinical applicability of coronary atherosclerotic lesion characterization. Minerva Cardioangiol 2011;59:255–70. 21516074

[2] Wirtz PH von Känel R. Psychological stress, inflammation, and coronary heart disease. Curr Cardiol Rep 2017;19:01–10. 10.1007/s11886-017-0919-x 28932967

[3] Kelly BB Narula J Fuster V. Recognizing global burden of cardiovascular disease and related chronic diseases. Mt Sinai J Med 2012;79:632–40. 23239202 10.1002/msj.21345

[4] Bhatt DL. Percutaneous coronary intervention in 2018. JAMA 2018;319:2127–8. 29800163 10.1001/jama.2018.5281

[5] Hamasaki S Tei C. Effect of coronary endothelial function on outcomes in patients undergoing percutaneous coronary intervention. J Cardiol 2011;57:231–8. 21398093 10.1016/j.jjcc.2011.02.003

[6] Lei W Li X Li L. Compound Danshen Dripping Pill ameliorates post ischemic myocardial inflammation through synergistically regulating MAPK, PI3K/AKT and PPAR signaling pathways. J Ethnopharmacol 2021;281:114438. 34390798 10.1016/j.jep.2021.114438

[7] Moher D Shamseer L Clarke M. Preferred reporting items for systematic review and meta-analysis protocols (PRISMA-P) 2015 statement. Syst Rev 2015;4:01–9. 10.1186/2046-4053-4-1 PMC4320440 25554246

[8] Higgins JP Altman DG Gøtzsche PC. The Cochrane Collaboration's tool for assessing risk of bias in randomised trials. BMJ (Clinical research ed) 2011;343:d5928. 10.1136/bmj.d5928 PMC3196245 22008217

[9] Lewis S Clarke M. Forest plots: trying to see the wood and the trees. BMJ (online) 2011;11:343–51. 10.1136/bmj.322.7300.1479 PMC1120528 11408310

[10] Borenstein M Hedges LV Higgins JP Rothstein HR. A basic introduction to fixed-effect and random-effects models for meta-analysis. Res Synth Methods 2010;1:97–111. 26061376 10.1002/jrsm.12

[11] Di Angelantonio E Thompson A Wensley F Danesh J. Coronary heart disease. IARC Sci Publ 2011;163:363–86. 22997872

[12] Lloyd-Jones DM Larson MG Beiser A Levy D. Lifetime risk of developing coronary heart disease. Lancet 1999;353:89–92. 10023892 10.1016/S0140-6736(98)10279-9

[13] Hoole SP Bambrough P. Recent advances in percutaneous coronary intervention. Heart 2020;106:1380–6. 32522821 10.1136/heartjnl-2019-315707

[14] Wu J Zhao L Lin K Lu L Luo C. Chinese herbal medicines for restenosis after percutaneous coronary intervention: a meta-analysis of randomized controlled trials. J Altern Complement Med 2019;25:983–92. 31464515 10.1089/acm.2018.0516

